# Defining and measuring food literacy in late childhood and adolescence: a scoping review

**DOI:** 10.1017/S1368980026102651

**Published:** 2026-05-15

**Authors:** Christine St Pierre, Rob M. van Dam, Jeffrey B. Bingenheimer, Jennifer M. Sacheck

**Affiliations:** 1 Department of Exercise and Nutrition Sciences, The George Washington University Milken Institute School of Public Health, USA; 2 Department of Prevention and Community Health, The George Washington University Milken Institute School of Public Health, USA; 3 Department of Social and Behavioral Sciences, Brown University School of Public Health, USA

**Keywords:** Food literacy, Concept, Measurement indicators, Children, Adolescents

## Abstract

**Objective:**

Food literacy is a multidimensional concept capturing interrelated factors driving individuals’ food behaviours. Conceptualisation to date has focused on adults without considering developmental limitations of childhood. This scoping review clarifies conceptualisation and measurement of food literacy in late childhood and adolescence.

**Design:**

We searched the literature in seven electronic databases, following the Preferred Reporting Items for Systematic Reviews and Meta-Analyses extension for Scoping Reviews for article screening and selection. We conducted an inductive content analysis to identify the primary dimensions and indicators of food literacy for children and adolescents and examined assessment of these dimensions in existing measurement tools.

**Setting:**

Articles covering late childhood and adolescent populations.

**Participants:**

Older children and adolescents (9–18 years).

**Results:**

The initial search in November 2023 yielded 1319 articles with thirteen meeting inclusion criteria, and a search update in February 2026 yielded four additional recently published articles for inclusion. We identified four dimensions and twenty-three reflective indicators of child and adolescent food literacy. Conceptualisation and measurement of food literacy in children to date have heavily emphasised dimensions pertaining to *food systems and nutrition knowledge* and *confidence in everyday food skills* relative to *valuing shared food experiences* and *purposeful engagement with food*.

**Conclusions:**

*Valuing shared food experiences* and *purposeful engagement with food* are understudied dimensions of food literacy, despite evidence that they are important determinants of food behaviours. Future research should focus on further conceptual development and validation of age-appropriate indicators for these dimensions.

Across the world, diets of children and adolescents are out of accord with dietary guidelines for an active, healthy life. Specifically, this population consumes too much energy-dense ultra-processed foods and sugar-sweetened beverages and too little fruits, vegetables and whole grains^(1,2)^. Dietary behaviours established during childhood tend to persist into adulthood^(3,4)^, and current patterns in children and adolescents’ dietary behaviours are associated with adverse health outcomes throughout the life course. High consumption of energy-dense foods and beverages is a driver of excess weight gain in childhood, which in turn has been linked to increased risk of cardiometabolic diseases and various types of cancer in adulthood^(5)^. Furthermore, among dietary factors, low fruit and whole grain intake have been identified as two of the largest contributors to chronic disease morbidity and mortality worldwide^(6)^.

Despite plentiful evidence for the connection between diet and health, understanding and addressing the complex interplay of factors that drive dietary behaviours has proven challenging. Studies with children and adolescents have identified sensory properties – especially taste, social desirability, perceived convenience, knowledge and skills as key drivers of food choice^(1,7,8)^. Knowledge in this context is not limited to scientific food and nutrition knowledge but also includes knowledge from lived experience in one’s individual and cultural food context^(9,10)^. Interventions aiming to improve children and adolescents’ dietary behaviours historically focused heavily on nutrition knowledge and food preparation skills, with limited evidence for sustained behaviour change^(11,12)^. In contrast, the food literacy field, which has emerged and rapidly evolved over the past 10–15 years, posits that effecting healthier dietary behaviours requires considering these driving factors in combination rather than in isolation^(13,14)^.

Food literacy is a multidimensional construct that was defined in its early emergence as the ‘relative ability to basically understand the nature of food and how it is important to you, and how able you are to gain information about food, process it, analyse it, and act upon it’^(15)^ Subsequent conceptualisation work emphasizes that individuals with high food literacy are empowered to make informed food decisions – towards individual, societal and environmental health – as they navigate the food system in their particular cultural, social and ecological context^(16–19)^. Assessment and monitoring of food literacy levels could be an important intermediate step in determining the most effective strategies for achieving improved dietary behaviours and ultimately, diet-related health outcomes, but the complex and expansive nature of the construct has been a barrier to finding consensus on its definition and dimensions^(16,20)^. Existing definitions have also been criticised for being too broad and vague to inform clear measurement indicators of food literacy as an individual-level variable^(21)^.

Findings from a 2021 review of existing food literacy measurement tools for children and adolescents indicated that food literacy frameworks have almost universally been developed in adult contexts, based on an assumption of agency over food choices, which children and adolescents only have to a limited degree^(22)^. One existing narrative review approached food literacy primarily in child and adolescent educational settings and developed one of the first food literacy frameworks extending beyond individual health, but several of the selected articles and the resulting framework were not specific to children and adolescents^(18)^. Authors of the measurement tool review emphasised the need for a food literacy definition and framework based on the unique developmental characteristics children and adolescents to inform future tools^(22)^. In 2023, a narrative review^(23)^ helpfully mapped food literacy competencies to children and adolescents’ developmental stages based on physiological and psychosocial development and progression of autonomy from childhood into adolescence^(24)^, but the food literacy conceptual framework used in the review^(25)^ was developed in a young adult population without the independence and agency limitations of children and adolescents.

Several reviews have sought to map the breadth of food literacy definitions, dimensions and indicators in adult-centred contexts to clarify conceptualisation and measurement of the construct^(17,26–28)^. To our knowledge, however, no review has done this same mapping for food literacy literature focused explicitly on older children and adolescents. In the present study, therefore, we aimed to review all the studies that have focused on conceptualisation and measurement of the full construct of food literacy in children and adolescents and from those, to identify consistent dimensions and reflective indicators that could contribute to a novel child and adolescent-centred framework.

## Methods

We conducted a scoping review, which allowed us to develop a conceptual framework to clarify understanding of the dimensions and indicators of food literacy as applied to older children and adolescents, as well as to determine the extent to which existing food literacy measurement tools assess these dimensions and identify current gaps between conceptualisation and measurement^(29)^. The review was conducted in accordance with the Joanna Briggs Institute (JBI) methodology for scoping reviews^(30)^, and findings were reported following the Preferred Reporting Items for Systematic Reviews and Meta-Analyses extension for Scoping Reviews (PRISMA-ScR) Checklist (Additional file 1)^(31)^. The review protocol was developed and registered with Open Science Framework prior to initiating the literature search.

### Search strategy

The original search was conducted in November 2023, and we searched seven bibliographic databases, including PubMed, CINAHL, ERIC, JSTOR, ProQuest Central, Scopus and ScienceDirect, for peer-reviewed papers published between 1 January 2001 and 31 October 2023. The selection of databases was deliberately broad to ensure we pulled publications from all disciplines that have contributed to the development of the construct of food literacy in children and adolescents. The start of the publication date range was set based on the first appearance of a definition of food literacy in the peer-reviewed literature in 2001^(32)^. We conducted an updated search in February 2026 to capture any papers published between 1 November 2023 and 31 January 2026. The search terms were selected in consultation with university library guides to be as comprehensive as possible in capturing the conceptualisation and measurement of food literacy in child and adolescent populations. As an example, the full electronic search strategy for ProQuest is available in the supplementary material.

### Screening and selection

Our inclusion criteria required articles to fully define the construct of food literacy, that is, to identify all dimensions included in their conceptualisation, not just list a few example attributes. Further, articles had to either describe or explore attributes/competencies of food literacy in late childhood or adolescence, and/or develop indicators to assess food literacy in this population. Articles that considered ‘food and nutrition’ literacy as a single concept were eligible for inclusion, but those that only considered nutrition literacy – which focuses specifically on nutrition information rather than the broader spectrum of food choice determinants^(33)^ – or that were restricted to any other subset of food literacy (e.g. food preparation literacy) were excluded. For this review, we were targeting conceptualisation of food literacy as latent variable whose level can be measured in individuals^(34)^. Articles that approached food literacy from a topical, curriculum or programme development perspective were therefore excluded unless they also presented a comprehensive theoretical framework or measurement tool for the target population. To align with the international context in which the concept of food literacy has evolved^(35)^, we did not restrict articles to particular countries.

We set the age range of focus from 9 to 18 years to capture developmental stages when children are growing in their capacity to make complex judgements about food^(24)^ but are not fully independent in their food choices. The lower bound of 9 years is the age at which the ability to provide consistent and reliable responses on questionnaires begins to emerge^(36)^, and the upper bound was set to exclude articles that considered older, post-secondary adolescents who experience a higher level of food choice agency and independence. Articles that defined and described food literacy over an age range that dipped below the lower bound were included, provided the upper bound was within 9 to 18 years.

Search results were imported into Covidence™, duplicates were removed and articles were reviewed independently by two members of the research team in two phases. In the title and abstract screening phase, articles were excluded if they (1) were not written in English, (2) did not address food literacy specifically, (3) focused on a population exclusively younger than 9 to 18 years old or included young adults beyond secondary school or (4) were not published in a peer-reviewed journal. Reference lists of full-text articles were reviewed for potentially relevant articles not captured in the literature search and added to full-text screening.

In the full-text screening phase, results were excluded if they: (1) were a stand-alone abstract rather than a full-text manuscript, (2) were found to meet the exclusion criteria from the title and abstract phase once the full text was reviewed, (3) did not define the complete construct of food literacy, (4) explicitly restricted their focus to a subset of food literacy attributes rather than furthering understanding of the construct as a whole, (5) applied the concept of food literacy to curriculum, programme or intervention components or (6) employed a previously validated food literacy measurement tool for evaluation rather than making a novel contribution to understanding food literacy conceptualisation and measurement. The review team documented the reason for each exclusion at the full-text stage. Conflicts in inclusion/exclusion decisions between the two reviewers were resolved by the first author.

### Data charting

Prior to beginning data charting, we classified the included articles into two groups: conceptual articles that defined and described food literacy attributes, and measurement articles that developed and tested food literacy assessment tools. Data charting spreadsheets and a charting guide were developed for each group and piloted by the research team. For all articles, we charted general study information, including author, title, publication year, study location (or country of affiliation at the time of publication for review authors), the age range of focus and demographic characteristics of any primary participants. We also extracted text passages that defined and described food literacy, peer-reviewed food literacy works cited in the definition/description, theoretical frameworks or models used to organise food literacy competencies or indicators, distinctions made between child food literacy and adult food literacy and the individual-level food literacy competencies or indicators identified in manuscript texts and tables. We extracted additional information from the measurement articles, including questionnaire format, response options and the final questionnaire items following psychometric testing.

### Data synthesis

To map the conceptual understanding of food literacy in older children and adolescents, we first conducted a descriptive numerical analysis^(37)^ to provide insight into the characteristics of the included articles, commonly cited food literacy definitions and use of theoretical frameworks or models. Food literacy is conceptualised as a multidimensional construct, but there is not universal agreement on the specific dimensions in adults, much less children and adolescents. We thus used inductive content analysis to identify the consistent dimensions and indicators appearing across the included articles. Following the inductive content analysis methodology described by Vears & Gillam^(38)^, two members of the research team first coded the extracted data to identify the overarching dimensions and then carried out a second round of coding to identify the properties – or in measurement terms, the reflective indicators^(39)^ of each dimension. The structure of the dimensions and their reflective indicators developed through the inductive content analysis was iteratively discussed and refined among the members of the research team until consensus was reached. We used a combination of deductive and inductive analysis approaches on the articles identified in our updated search, coding the data using the dimensions and reflective indicators that had already been identified. We also allowed for the possibility of new codes emerging inductively but did not find any new dimensions or indicators.

To account for potential expansion of reflective indicators as children progress developmentally, we developed a matrix visualisation of the frequency with which each reflective indicator was included at each of the three developmental stages covered by the included articles: late childhood, early adolescence and late adolescence. The developmental stages were connected to schooling level, as there are discernable differences by level in school environments, curriculum content and education approaches in most countries, while age ranges may overlap slightly across stages. Late childhood refers to the upper elementary or primary grades (about 9–12 years), early adolescence refers to middle/junior high school or lower secondary school (about 12–15 years) and late adolescence refers to high school or upper secondary school (about 15–18 years). In articles that spanned multiple developmental stages, we included them in the matrix at all applicable stages.

We examined the extent to which existing tools measured each of the identified dimensions by mapping the questionnaire items to the dimensions and calculating the proportion of items in the questionnaire assessing each respective dimension. To identify gaps in food literacy measurement among our target population, we compared the relative proportion of questionnaire items assessing each dimension and identified dimensions that are currently understudied.

## Results

### Article characteristics

**Figure 1.**
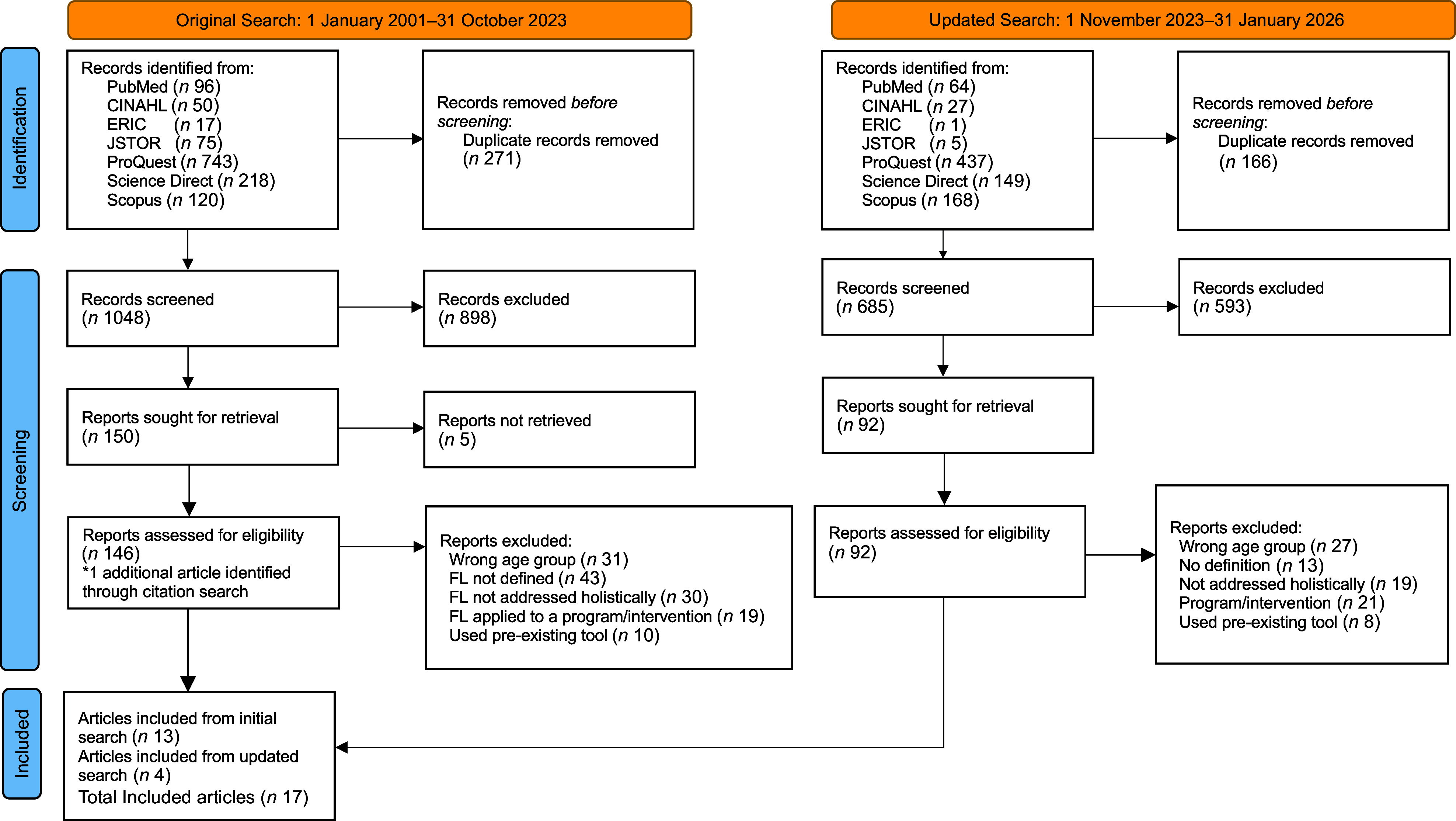
Figure 1 long description. PRISMA flow diagram: defining and measuring food literacy in late childhood and adolescence.

Our initial search yielded 1319 articles after duplicates were removed. After the two rounds of review, thirteen articles met our eligibility criteria – seven conceptual^(21,23,40–44)^ and six measurement^(45–50)^ articles (Figure 1). The updated search yielded four additional measurement articles published between 2024 and 2026^(51–54)^. The study characteristics are summarised in Table 1. Fifteen of the articles included primary research conducted in twelve different countries: Australia (*n* 1), Canada (*n* 1), China (*n* 1), Denmark (*n* 1), France (*n* 1), India (*n* 1), Iran (*n* 2), the Netherlands (*n* 1), Peru (*n* 1), South Korea (*n* 1), Thailand (*n* 1) and the USA (*n* 2). One narrative review was included, with authors representing Uruguay, France, the Netherlands, Ireland, the UK and Norway, and one commentary was included by authors from Canada.

**Table 1.** Characteristics of included studies Table 1 long description.

Study details	Country	Article type	Age range/ developmental stage	Participant characteristics	Peer-reviewed works referenced in food literacy definition	Organising frameworks or indicators	Food literacy levels/domains	Child/adolescent- specific food literacy considerations
Conceptual								
Amin, 2018^(40)^	USA	Research: qualitative	9–12 years old/late childhood	*n* 31 Urban 65 % female 43 % economically disadvantaged	Murimi, 2013 Vidgen & Gallegos, 2014	Vidgen & Gallegos, 2014	*Non-hierarchical:* Planning & management Selection Preparation Eating	Indicators match developmental stage Limited agency affects indicators
Ares, 2023^(23)^	Uruguay, France, Netherlands, Ireland, UK and Norway	Narrative review	Late childhood^*^ Early adolescence Late adolescence	NA	Cullen *et al.*, 2015 Pope *et al.*, 2021 Sumner, 2015 Truman *et al.*, 2017	Nutbeam, 2000 Slater *et al.*, 2018	*Hierarchical:* Relational competencies Functional competencies Critical competencies	Indicators match developmental stage Limited agency affects indicators Children can influence household through food literacy
Doustmohammadian, 2022^(41)^	Iran	Research: conceptual framework development	10–12 years old/late childhood	*n* 89 Urban 47 % female About 33 % economically disadvantaged	Vidgen & Gallegos, 2014	Nutbeam, 2000	*Hierarchical:* Functional skills Interactive skills Critical skills	Indicators match developmental stage
Lam, 2019^(42)^	Canada	Research: qualitative	Late adolescence	*n* 16 Urban 56 % female (SES not reported)	Renwick & Powell, 2019 Renwick, 2017	Renwick, 2017	*Non-hierarchical:* Operational Cultural Critical	
Ronto, 2016^(43)^	Australia	Research: mixed methods	12–17 years/ Early adolescence Late adolescence	*n* 131 (Urbanicity not reported) 69 % female (SES not reported)	Colatruglio & Slater, 2016 Cullen *et al.*, 2015 Fordyce-Voorham, 2011 Smiith, 2009 Sumner, 2013 Vaitkeviciute *et al.*, 2015 Vidgen & Gallegos, 2014	Developed a list of twenty-two food literacy aspects drawing from published literature	*Non-hierarchical:* Food & nutrition knowledge Food skills Capacity	Limited agency affects indicators Children can influence household through food literacy
Samruayruen, 2022^(44)^	Thailand	Research: qualitative	12–15 years/ Early adolescence	*n* 17 (Urbanicity not reported) 88 % female (SES not reported)	Cullen *et al.*, 2015 Vaitkeviciute *et al.*, 2015	Nutbeam, 2000	*Hierarchical:* Functional skills Interactive skills Critical skills	Indicators match developmental stage Children can influence household through food literacy
Truman, 2017^(21)^	Canada	Commentary	Late childhood Early adolescence Late adolescence^†^	NA	Developed a consensus definition through stakeholder meetings	None	NA	Indicators match developmental stage
Measurement								
Amin, 2019^(45)^	USA	Research: scale development	9–11 years/ Late childhood	*n* 706 Suburban (Sex not reported) 26–65 % economically disadvantaged	Truman *et al.*, 2017 Vidgen & Gallegos, 2014	Vidgen & Gallegos, 2014 Amin, 2018	*Non-hierarchical:* Cooking skills Cooking knowledge Nutrition knowledge Food systems knowledge Self-efficacy around eating	
Ashoori, 2020^(46)^	Iran	Research: scale development	17–18 years/ Late adolescence	*n* 697 Urban 56 % female 23 % economically disadvantaged	Vidgen & Gallegos, 2014	Nutbeam, 2000 Doustmohammadian, 2022	*Hierarchical:* Knowledge Functional literacy Interactive literacy Advocacy Critical literacy Food label literacy	
Doustmohammadian, 2017^(47)^	Iran	Research: scale development	10–12 years/late childhood	*n* 373 Urban 49 % female About 33 % economically disadvantaged	Vidgen & Gallegos, 2014	Nutbeam, 2000 Doustmohammadian, 2022	*Hierarchical:* Understanding Knowledge Functional literacy Interactive literacy Food choice Critical literacy	Indicators match developmental stage
Liu, 2021^(48)^	China	Research: scale development	13–15 years/ Early adolescence^‡^	*n* 2452 62 % rural registered residence 50 % female 14 % economically disadvantaged	Azevedo Perry *et al.*, 2017 Krause *et al.*, 2018 Vidgen & Gallegos, 2014	Nutbeam, 2000	*Hierarchical:* Knowledge and understanding Accessing and planning skills Selection skills Preparation skills Eating skills	
Park, 2022^(49)^	South Korea	Research: Scale development	13–18 years/ Early adolescence Late adolescence	*n* 200 Urban 48 % female (SES not reported)	Vidgen & Gallegos, 2014 Yuen *et al.*, 2018	Nutbeam, 2000 Park *et al.*, 2020	*Hierarchical:* Selection Production Distribution Preparation and cooking Intake	Indicators match developmental stage Limited agency affects indicators
Stjernqvist, 2021^(50)^	Denmark	Research: scale development	12–14 years/early adolescence	*n* 817 (Urbanicity not reported) 55 % female (SES not reported)	Benn, 2014	Benn, 2014	*Non-hierarchical:* To know To do To sense To care To want	Indicators match developmental stage Children can influence household through food literacy
Dallant, 2024^(51)^	France	Research: scale development	8–11 years/late childhood	*n* 1187 (Urbanicity not reported) 52 % female 55 % from priority education area	Azevedo Perry *et al.*, 2017 Cullen *et al.*, 2015	Azevedo Perry *et al.*, 2017	*Non-hierarchical:* Food and nutrition knowledge Food skills Self-efficacy and confidence Ecologic Food decisions	Children can influence household through food literacy
Yadav, 2025^(52)^	India	Research: scale development	13–15 years/early adolescence	*n* 400 Urban (Sex not reported) (SES not reported)	Krause *et al.*, 2018	Nutbeam, 2000 Doustmohammadian, 2022	*Hierarchical:* Functional literacy Interactive literacy Critical literacy	Indicators match developmental stage
Ponce-Carreón, 2025^(53)^	Peru	Research: scale development	10–13 years/late childhood	*n* 203 Urban About 50 % female (SES not reported)	Vidgen & Gallegos, 2014	Nutbeam, 2000 Slater *et al.*, 2018	*Hierarchical:* Relational competencies Functional competencies Critical competencies	Indicators match developmental stage
van Lier, 2026^(54)^	The Netherlands	Research: scale development	8–12 years/late childhood	*n* 608 (Urbanicity not reported) 38 % female (SES not reported)	Murimi, 2013 Vidgen & Gallegos, 2014 Velardo, 2015	European Commission Farm to Fork Strategy	*Non-hierarchical:* Food production Food processing and distribution Food consumption Food loss and waste prevention	Indicators match developmental stage Limited agency affects indicators

* Article also conceptualised food literacy in early and middle childhood, which was outside the scope of this review.

† Article did not distinguish between different developmental stages.

‡ Developmental stage of focus for psychometric testing.

Among the conceptual articles, one looked at food literacy in children and adolescents broadly, without distinguishing between developmental stages^(21)^, and one differentiated between children and adolescents in development of food literacy^(23)^. The remaining five conceptual articles included qualitative or mixed methods research with children or adolescents. Two focused specifically on late childhood^(40,41)^, one on early adolescence^(44)^, one on late adolescence^(42)^ and one included participants spanning both early and late adolescence^(43)^. The proportion of participants identifying as female ranged from 47 % to 88 % across the five studies. Participants were recruited from specific urban settings in Canada, Iran and the USA and from across a particular state or province in Australia and Thailand, respectively. The two studies reporting socio-economic indicators^(40,41)^ indicated diversity in socio-economic status among participants within their respective country context.

Among the ten food literacy measurement studies, five instruments targeted late childhood^(45,47,51,53,54)^, three focused on early adolescence^(48,50,52)^, one targeted late adolescence (17–18 years)^(46)^ and one spanned both early and late adolescence^(49)^. Five of the instruments were tested with children and adolescents in urban settings^(46,47,49,52,53)^, one in a suburban setting^(45)^ and four in settings with either varying or unspecified levels of urbanicity^(48,50,51,54)^. Studies reported recruiting students from diverse socio-economic backgrounds, and the proportion of participants identifying as female ranged from 38 % to 56 %.

#### Definitions and frameworks referenced

The articles in this review referenced eighteen different general food literacy definitions from the peer-reviewed literature^(11,17–19,26,27,33,55–65)^ when conceptualising food literacy for children and adolescents. Food literacy was described in all articles as a multidimensional concept encompassing the attributes that enable individuals to make informed food decisions within their food environment and context. A majority of articles (*n* 10, 59 %) emphasised that food literacy goes beyond facilitating food choices for personal health to include an understanding of all components of the food system, leading to a consideration of the social and environmental implications of food choices^(21,23,40,42,43,45,49–51,54)^. Six of the articles described or measured food and nutrition literacy together, and these had a noticeably stronger emphasis on individual health than on the food system^(41,44,46–48,52)^.

The most common underlying framework cited in the included articles (*n* 8, 47 %) was Nutbeam’s hierarchical health literacy model. Nutbeam’s model posits that health literacy levels build on one another from functional (basic knowledge and skills) to interactive (higher-level communication and social skills) to critical (analysis and application skills to advocate for oneself and make informed decisions)^(66)^. Six of the eight articles transferred this model directly from health literacy to food literacy, where the functional level included knowledge and skills related to the daily activities of selecting, preparing and eating food; the interactive level included seeking reliable sources of food and nutrition information and forming food practices through discussion and interaction with others; and the critical level included evaluating the quality of food and nutrition information, recognising food marketing tactics and active engagement in promoting health and sustainable food systems^(41,44,46–48,52)^. One study integrated the three literacy levels from Nutbeam’s model with eight components of the food system (production, processing, distribution, planning and management, selection, preparation and cooking, intake and disposal)^(67)^, to produce a two-dimensional conceptual model for food literacy^(49)^. One article^(23)^ built on Nutbeam’s model but also incorporated Slater’s framework of food literacy competencies^(25)^. In this conceptualisation, the hierarchy of food literacy levels was aligned with child development progression, where relational (rather than interactive) competencies develop first, followed by functional, and then critical. One of the measurement tools identified in the updated search then used this Nutbeam–Slater combined framework^(53)^

Five non-hierarchical models were the foundation for conceptualising food literacy in one article each: Vidgen & Gallegos’^(19)^ 4-domain model (plan & manage, select, prepare, eat), Benn’s^(18)^ 5-domain model (to know, to do, to sense, to care, to want), Renwick’s^(59)^ 3D model of critical literacy (operational, cultural and critical literacies), Azevedo-Perry’s^(26)^ 5-category framework (food and nutrition knowledge, food skills, self-efficacy and confidence, ecologic and food decisions) and the European Commission’s^(68)^ Farm to Fork Strategy (food production, food processing and distribution, food consumption, and food loss and waste prevention). The remaining three articles did not include any underlying frameworks or models. In three cases, the frameworks developed in conceptualisation articles included in the review^(40,41)^ informed subsequent development of measurement tools that were also included^(45,47,52)^.

#### Child and adolescent-specific considerations

Fourteen of the included articles (82 %) identified particular considerations when conceptualising food literacy in children and adolescents *v*. adults. The most common consideration was developmental changes through childhood and adolescence that necessitate the development of food literacy indicators appropriate to each developmental stage^(21,23,40,41,44,45,47,49,50,52–54)^. Additionally, some articles noted that limited agency over food decisions and practices in the home may render some common indicators of food literacy in adults irrelevant for children and adolescents^(23,40,43,49,54)^. Finally, several articles noted that high food literacy could help children positively influence their household’s food practices, even if they are not the primary decision-makers^(23,43,44,50,51)^.

### Dimensions of food literacy

Through our inductive content analysis, we identified four overarching dimensions of food literacy across the included articles: (1) food systems and nutrition knowledge, (2) confidence in everyday food skills, (3) valuing shared food experiences and (4) purposeful engagement with food. We also found twenty-three reflective indicators of food literacy that were consistently repeated over the majority of articles. Table 2 shows the matrix of the identified food literacy dimensions, their reflective indicators and the number and proportion of articles at each developmental stage (late childhood (*n* 9), early adolescence (*n* 8) and late adolescence(*n* 6) that included the respective indicators. If articles spanned multiple developmental stage, they are included in the *n* for each applicable stage.

**Table 2.** Matrix of number and proportion of articles including food literacy dimensions and indicators by developmental stage Table 2 long description.

Dimensions and indicators	Late childhood *n* 9 articles		Early adolescence *n* 8 articles		Late adolescence *n* 6 articles	
	*n*	%	*n*	%	*n*	%
Food systems and nutrition knowledge	9	100 %	8	100 %	6	100 %
Knowledge of food origins	8	≥ 75 %	6	≥ 75 %	6	100 %
Knowledge of food processing and supply chains	7	≥ 75 %	5	≥ 50 %	4	≥ 50 %
Recognise sustainable practices in the food system	2	< 25 %	5	≥ 50 %	6	100 %
Knowledge of food groups and dietary guidelines	7	≥ 75 %	7	≥ 75 %	4	≥ 50 %
Understand connections between food, nutrition and health	9	100 %	8	100 %	6	100 %
Understand information provided on food labels	8	≥ 75 %	7	≥ 75 %	4	≥ 50 %
Recognise food advertising/marketing strategies	3	≥ 25 %	5	≥ 50 %	3	≥ 50 %
Identify credible and reliable food and nutrition information	5	≥ 50 %	7	≥ 75 %	5	≥ 75 %
Confidence in everyday food skills	9	100 %	8	100 %	6	100 %
Able to make informed food selections in different settings (e.g. home, school, store, restaurant)	6	≥ 50 %	4	≥ 50 %	3	≥ 50 %
Able to practice safe food handling and proper hygiene	8	≥ 75 %	7	≥ 75 %	5	≥ 75 %
Able to use kitchen tools and appliances	4	≥ 25 %	4	≥ 50 %	2	≥ 25 %
Able to follow and adapt recipes	2	< 25 %	4	≥ 50 %	4	≥ 50 %
Able to prepare and/or cook different dishes	8	≥ 75 %	7	≥ 75 %	6	100 %
Able to plan and budget for healthy food intake	3	≥ 25 %	3	≥ 25 %	3	≥ 50 %
Able to practice strategies to reduce food waste (e.g. use leftovers, compost, recycle)	1	< 25 %	3	≥ 25 %	2	≥ 25 %
Able to grow food through gardening	2	< 25 %	3	≥ 25 %	3	≥ 50 %
Valuing shared food experiences	9	100 %	8	100 %	6	100 %
Valuing shared meals	3	≥ 25 %	3	≥ 25 %	3	≥ 50 %
Valuing participation in household food activities (e.g. shopping for food, preparing/cooking food)	3	≥ 25 %	4	≥ 50 %	3	≥ 50 %
Appreciating/understanding cultural food practices and traditions	3	≥ 25 %	5	≥ 50 %	4	≥ 50 %
Valuing sharing food and nutrition information with family and friends	4	≥ 25 %	5	≥ 50 %	3	≥ 50 %
Purposeful engagement with food	8	≥ 75 %	7	≥ 75 %	5	≥ 75 %
Willing to try new foods	4	≥ 25 %	4	≥ 50 %	3	≥ 50 %
Consciously enjoy eating a variety of foods	2	< 25 %	5	≥ 50 %	3	≥ 50 %
Deliberate sensory and/or mind-body awareness when engaging with food	8	≥ 75 %	4	≥ 50 %	3	≥ 50 %

#### Food systems and nutrition knowledge

Knowledge was the most developed dimension of food literacy and was present in all the articles. This dimension covers a wide breadth of topics, including the food system from farm to plate, as well as individual nutrition and health. Understanding connections between food, nutrition and health was universally cited as a reflective indicator in this dimension among the included articles, and knowledge of food origins was an indicator in all but three of the articles^(44,52,53)^. The knowledge dimension also indicated progression as children grow older: knowledge of simple food processing and supply chain steps were emphasised in late childhood, while evaluating the sustainability of food system practices was a more common indicator for adolescents. Similarly, understanding the information provided on food labels was repeated at all developmental stages, while recognition of food advertising/marketing strategies and identifying credible sources of food and nutrition information – indicators that require higher-order critical thinking – were found more often in the articles focused on adolescents.

#### Confidence in everyday food skills

This dimension focuses on a person’s perception of their abilities or skill level in carrying out the day-to-day activities required to feed themselves and was present in all articles. The skills identified in this dimension did not readily lend themselves to objective measurement; thus, the reflective indicators rely on self-assessment of skills or abilities. The ability to prepare and/or cook different dishes and the ability to practice safe food handling were both nearly universal indicators in this dimension. Additional frequently repeated indicators were the ability to make informed food choices in different settings, the ability to follow or adapt recipes and the ability to use kitchen tools and appliances. Indicators requiring more advanced skills – including planning and budgeting for healthy food intake, practicing strategies to reduce food waste and growing food/gardening – were more commonly repeated in articles focused on adolescence compared to those focused on late childhood.

#### Valuing shared food experiences

The ‘valuing shared food experiences’ dimension captures the social and relational aspects of food literacy identified across all included articles. This dimension recognises that food behaviours are not shaped in isolation but through interaction and conversation in community, particularly with family and friends. The most repeated reflective indicators were valuing shared meals and sharing food and nutrition information with family and friends. Valuing participation in household food activities and appreciating cultural food practices/traditions were included as indicators in more than half of the articles and were more prevalent in those focused on adolescents. Across all developmental stages, child and adolescent study participants contributing to food literacy conceptualisation identified shared meals and participation in household food activities as both enjoyable and important aspects of their interaction with food^(40,42,43)^.

#### Purposeful engagement with food

This dimension covers the level of physical, sensory, mental and emotional awareness and intention an individual possesses when interacting with food (e.g. learning about new foods, preparing food, etc.) and when eating. Purposeful engagement with food was the least clearly developed dimension, but it was identified in most of the included articles (*n* 14, 82 %) and was distinct from the other three identified dimensions. Sensory and/or mind–body awareness when engaging with food was the most referenced indicator, and a willingness to try new foods was also common across all developmental stages. Conscious enjoyment of a variety of foods was a more common indicator for adolescence than late childhood, but overall, this dimension was more heavily emphasised at younger developmental stages. The dimension and its indicators were also described more thoroughly in the conceptual articles relative to the measurement articles.

### Measurement of food literacy dimensions

The six measurement tools included in the review ranged in length from 19 to 73 items. While Likert-type response scales based on the respondent’s subjective perspective were the most common format for the items across all tools, nine included some multiple choice, true/false or matching items with objectively correct or incorrect answers^(45–48,50–54)^. Two of the tools focused on the late childhood developmental stage and one focused on early adolescence were completely^(52,54)^ or mostly (82 %)^(45)^ comprised of items with objectively correct or incorrect answers. Likert-type items assessing level of agreement with questionnaire statements made up half or more of the items in six of the tools^(46,47,49–51,53)^. Five tools were designed for late childhood^(45,47,51,53,54)^, three for early adolescence^(48,50,52)^, one for late adolescence^(46)^ and one spanned both early and late adolescence^(49)^. Table 3 summarises characteristics of the measurement tools and identifies the proportion of items assessing each of the four identified food literacy dimensions. In every tool, most items assessed food and nutrition knowledge and confidence in everyday food skills, and in nine of the ten tools, 70 % or more of questionnaire items were devoted to these two dimensions.

**Table 3.** Characteristics of child or adolescent food literacy tools and proportional assessment of identified food literacy dimensions Table 3 long description.

	TFLAC^(35)^	FNLIT^(37)^	Dallant *et al.*^(51)^	Ponce-Carreón *et al.*^(53)^	DCFLQ^(54)^	FNLQ-SC^(38)^	FLQ-sc^(40)^	INFOLIT^(52)^	Park *et al.*^(39)^	FNLAT^(36)^
Characteristics										
Number of questionnaire items	40*	42	25	20	42	48*	37	73	19	60
Proportion of questionnaire items with objectively correct response options	82 %	17 %	48 %	35 %	100 %	21 %	5 %	100 %	0 %	50 %
Developmental stage(s) of focus	LC	LC	LC	LC	LC	EA	EA	EA	EA, LA	LA
Proportion of questionnaire items assessing identified dimensions^†^										
Food systems and nutrition knowledge	55 %	31 %	36 %	45 %	81 %	37 %	22 %	64 %	32 %	63 %
Confidence in everyday food skills	35 %	38 %	44 %	50 %	14 %	44 %	32 %	29 %	47 %	38 %
Valuing shared food experiences	5 %	19 %	8 %	5 %	5 %	15 %	24 %	3 %	11 %	8 %
Purposeful engagement with food	5 %	12 %	12 %	0 %	0 %	4 %	22 %	4 %	11 %	0 %

TFLAC, Tool for Food Literacy Assessment in Children (Amin *et al.*); FNLIT, Food and Nutrition Literacy scale (Doustmohammadian *et al.*); DCFLQ, Dutch Children’s Food Literacy Questionnaire (van Lier *et al.*); FNLQ-SC, Food and Nutrition Literacy Questionnaire for Chinese School-age Children (Liu *et al.*); FLQ-sc, Food Literacy Questionnaire designed for schoolchildren (Stjernqvist *et al.*); FNLAT, Food and Nutrition Literacy Assessment Tool (Ashoori *et al.*); LC, late childhood; LA, late adolescence; EA, early adolescence.

* Number of unique scored items, including unnumbered subquestions.

† Proportions may not add to 100 % due to rounding.

#### Current measurement gaps

The ‘valuing shared food experiences’ and ‘purposeful engagement with food’ dimensions were much less frequently measured in existing tools compared with the other two dimensions. Although every tool assessed some aspect of the ‘valuing shared food experiences’ dimension, only one tool included items assessing all four of the dimension’s reflective indicators^(50)^. The ‘purposeful engagement with food’ dimension was proportionally the least frequently assessed dimension, and three tools did not include items for any of the indicators in this dimension^(46,53,54)^.

## Discussion

This review examined and synthesised the construct of food literacy as it applies to individuals in late childhood and adolescence and identified the extent to which existing measurement tools assess the identified dimensions of food literacy in this population. Across all included articles, food literacy was characterised as both a complex, multidimensional concept and a potentially important lever for empowering children and adolescents to make informed, healthy and sustainable food decisions. While there was no clear consensus on a single definition or conceptual framework for food literacy in children and adolescents, we inductively identified four primary food literacy dimensions that were consistently repeated across the articles: food and nutrition knowledge, confidence in everyday food skills, valuing shared food experiences and purposeful engagement with food.

A major motivation for undertaking this review was to develop a food literacy framework to guide questionnaire development that is appropriate for the limited food choice agency and ongoing cognitive development of children and adolescents. Many items in adult food literacy assessment tools focus on actions like food selection, purchasing and preparation decisions that children and adolescents most likely do not control in their households^(69–72)^. Even within the studies included here that focus specifically on children and adolescents, most of the underlying food literacy conceptual frameworks (*n* 11, 65 % of included studies) have a ‘critical literacy’ dimension that is beyond the developing critical thinking and analytical skills of this population^(73)^. Indeed, in child and adolescent measurement tools that included a ‘critical’ dimension, item factor loadings and internal consistency tended to be lower relative to other dimensions^(46,47,50)^. Our inductive approach allowed for viewing these works through a new lens and led to identification of dimensions that can remain consistent through childhood and adolescence. Basic, foundational indicators for these dimensions can be the focus in late childhood, with more analytical indicators increasing through adolescence.

Further development and study of the ‘valuing shared food experiences’ and ‘purposeful engagement with food’ dimensions may be particularly important for understanding food literacy in children and adolescents. These findings align with previous calls for a greater emphasis on the social aspects of food and eating within the study of food literacy^(56,74)^.Previous food behaviour research with this population has indicated that whether as a facilitator or barrier, social connection and relationships – with family and peers – are a primary determinant of healthy food choices^(75,76)^. Similarly, food preferences are a major driver of food decisions for children and adolescents^(7,77)^, and the sensory appeal of foods is the primary factor in shaping food desirability for this population^(78)^ – findings that support the importance of ‘purposeful engagement with food.’ Despite the evidence for these dimensions as determinants of food behaviours in children and adolescents, all but one of the measurement tools^(50)^ devoted less than one-third of items to assessing them. There is a critical need to explore these dimensions further and develop age-appropriate indicators for each to build the evidence base of best practices for improving children’s dietary behaviours and their subsequent health outcomes.

Food-related knowledge and skills, which have been a primary focus in adult food literacy^(13,20,79)^, were confirmed as key dimensions of child and adolescent food literacy. However, these dimensions may currently be overemphasised in food literacy measurement tools relative to their contribution to food behaviours. Although adolescents themselves have identified knowledge as important for making healthy food choices^(43,77)^, research also indicates that knowledge alone is insufficient to change food behaviour^(11,12)^. Furthermore, a lack of confidence in everyday food skills was identified in one of the included articles as a barrier to children and adolescents putting knowledge into practice^(43)^. This aligns with existing research indicating that beyond the skills themselves, confidence or self-efficacy regarding one’s cooking ability plays a role in food choices^(80–82)^. Greater understanding of how the four food literacy dimensions interact with one another and contribute to children’s food decisions can inform the appropriate weighting of each dimension in future development of food literacy measurement tools for this population.

To our knowledge, this is the first scoping review to map the existing literature on conceptualisation and measurement of food literacy in late childhood and adolescence. Our review helps to clarify the scope of food literacy in this population by identifying four primary dimensions of food literacy and connecting indicators for each dimension to the developmental stages of late childhood and adolescence. The synthesis of existing food literacy measurement tools for this population and identification of current gaps provides guidance for future tool development to monitor and evaluate food literacy in children. An additional strength of this review is the inclusion of studies from a wide variety of settings worldwide, suggesting that our findings on the key dimensions of food literacy are broadly applicable in child and adolescent populations.

This study should also be considered in the context of its limitations. In line with standard scoping review methods, we did not appraise and thus cannot speak to the quality of evidence from included studies. Restriction to English-language articles coupled with the context-specific nature of food environments limits the generalisability of our findings, despite consistency across the diverse settings represented in our included articles. It could be argued that the author overlap in two pairs of conceptual and measurement articles biases the consistency of results, but we included both types of articles because there were multiple indicators conceptualised but not measured, which aided in mapping the concordance between conceptualisation and measurement. Lastly, there is inherent subjectivity in conceptualising food literacy, and the four dimensions we identified through our content analysis may not capture all salient aspects of food literacy in children and adolescents. Our review, however, does offer a comprehensive synthesis of the conceptualisation and measurement of food literacy in children and adolescents to date and identifies future directions for research.

## Conclusion

Understanding food literacy in children and adolescents has the potential to shape food education and experiences that more effectively promote the development of healthy dietary behaviours in this population. We provide a synthesis of four dimensions of child and adolescent food literacy and the reflective indicators of each dimension identified in the existing literature. This enhanced understanding of food literacy can inform the development of age-appropriate food education programmes and interventions that help children and adolescents develop food behaviours associated with better health outcomes throughout the life course. Future research should focus on further development of the ‘valuing shared food experiences’ and ‘purposeful engagement with food’ dimensions of food literacy and on generating more empirical data to inform robust approaches to measurement at each developmental stage of childhood and adolescence.

## Supporting information

10.1017/S1368980026102651.sm001 St Pierre et al. supplementary material St Pierre et al. supplementary material

## Supplementary material

For supplementary material accompanying this paper visit https://doi.org/10.1017/S1368980026102651

## Figure 1. Long description

The flowchart begins with the identification phase, where records are identified from various databases such as PubMed, CINAHL, ERIC, JSTOR, ProQuest, Science Direct, and Scopus. For the original search from January 1, 2001, to October 31, 2023, a total of 1279 records are identified. After removing 271 duplicate records, 1048 records are screened, and 898 are excluded. 150 reports are sought for retrieval, with 5 not retrieved. 146 reports are assessed for eligibility, and 1 additional article is identified through citation search. Reports are excluded for various reasons such as wrong age group, undefined food literacy, not addressed holistically, and use of pre-existing tools. 13 articles are included from the initial search. For the updated search from November 1, 2023, to January 31, 2026, 757 records are identified. After removing 166 duplicate records, 685 records are screened, and 593 are excluded. 92 reports are sought for retrieval, with none not retrieved. 92 reports are assessed for eligibility, and 4 articles are included from the updated search. The total number of included articles is 17.

Navigate back to Figure 1..

## Table 1. Long description

The table presents a detailed summary of studies focused on child development and nutrition, including conceptual and measurement articles. It lists studies from twelve different countries, such as Australia, Canada, China, Denmark, France, India, Iran, the Netherlands, Peru, South Korea, Thailand, and the USA. The table includes columns for study details, country, article type, age range, and various other relevant factors. Each row provides specific information about individual studies, such as the study design, participant characteristics, and key findings. The table highlights the diversity of research methods and focuses across different regions and contexts.

Navigate back to Table 1..

## Table 2. Long description

The table presents a matrix of food literacy dimensions and indicators across three developmental stages: late childhood, early adolescence, and late adolescence. It includes data from nine articles for late childhood, eight for early adolescence, and six for late adolescence. The table is structured with dimensions and indicators as rows and the developmental stages as columns, showing the number and proportion of articles that include each indicator. Key dimensions include food systems and nutrition knowledge, confidence in everyday food skills, valuing shared food experiences, and purposeful engagement with food. Notable trends include high proportions of articles addressing food systems and nutrition knowledge and confidence in everyday food skills across all stages. The table highlights the consistency and variability of food literacy indicators across different developmental stages.

Navigate back to Table 2..

## Table 3. Long description

The table compares characteristics of child or adolescent food literacy tools and proportional assessment of identified food literacy dimensions. It includes data from ten different tools, detailing the number of questionnaire items, the proportion of items with objectively correct response options, the developmental stages of focus, and the proportion of items assessing four identified food literacy dimensions. The tools range in length from 19 to 73 items. The table highlights that two tools focus on the late childhood developmental stage and one on early adolescence, with a high proportion of items having objectively correct or incorrect answers. Likert-type items assessing level of agreement with questionnaire statements make up half or more of the items in six of the tools. The table also shows that most items in every tool assess food and nutrition knowledge and confidence in everyday food skills, with nine of the ten tools devoting 70% or more of their items to these two dimensions.

Navigate back to Table 3..
