# Supplementary material for: Defining and measuring food literacy in late childhood and adolescence: a scoping review
Source: Public Health Nutr. 2026 May 15;29(1):e111. doi: 10.1017/S1368980026102651 (PMC13434494; doi:10.1017/S1368980026102651)
Supplement: Supplementary file 1 [file S1368980026102651sup001.docx]

**Food literacy in children and adolescents scoping review: search terms and strategy**

*Supplementary Table 1: Search terms*

| **Topic** | **Concept** | **Population** |
| --- | --- | --- |
| Food literacy | Definition  Concept*  Model  Framework  Dimension*  Domain*  Theor*  Skill*  Attribute*  Competenc* | Child*  Adolesc*  Youth |

*Supplementary Table 2: Example search strategy (ProQuest)*

Advanced Search – Command Line

“food literacy” AND

definition OR concept* OR model OR framework OR dimension* OR domain* OR theor* OR skill* OR attribute* OR competenc* AND

child* OR adolesc* OR youth

Limit to: Peer reviewed

Publication date:

Start: January 1, 2001

End: October 31, 2023

Language: English
